# Cutaneous metastasis on the nasal tip: first clinical sign of pulmonary carcinoid tumor^☆^

**DOI:** 10.1016/j.abd.2021.03.002

**Published:** 2021-07-18

**Authors:** Lara Martins Fiorio, Lucia Martins Diniz, Elton Almeida Lucas

**Affiliations:** a Hospital Universitário Cassiano Antônio de Moraes, Vitória, ES, Brazil; b Universidade Federal do Espírito Santo, Vitória, ES, Brazil

**Keywords:** Carcinoma, neuroendocrine, Neoplasm metastasis, Neoplasms, unknown primary

## Abstract

Skin metastases are rare and may occur in the context of a known metastatic disease or be the first clinical sign of an underlying primary tumor. In the case of carcinoid neoplasms, determining whether the cutaneous tumor is primary or secondary and identifying the tumor origin in metastatic cases is not always an easy task. This is the report of a case of cutaneous metastasis presenting as the first clinical manifestation of a previously unknown pulmonary carcinoid tumor, including the discussion of histopathological and immunohistochemical findings that allowed an adequate diagnosis of the tumor etiology and reinforces the importance for dermatologists and dermatopathologists to be familiar with these findings.

## Introduction

Skin metastases are a rare clinical finding, with an incidence between 0.7% and 10% in cancer patients and represent only 2% of all skin tumors.^1^ They usually occur in a clinical setting of known generalized metastatic disease; however, they can be the first sign of an underlying silent neoplasm, as well as an early sign of the recurrence of a previously. Because of this, they usually indicate a poor prognosis and the mean survival of patients with metastatic cutaneous disease is 7.5 months.^2^ This is the report of a patient with a metastatic skin lesion that presented as the first manifestation of a pulmonary carcinoid tumor.

## Case report

**Figure 1:**
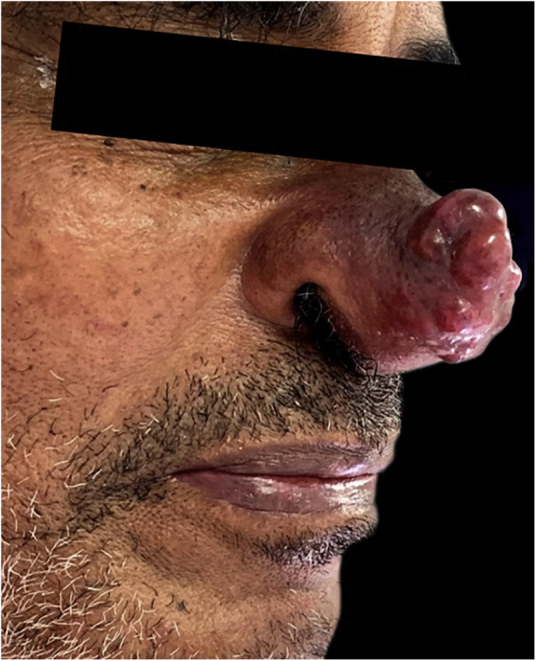
Exophytic, painful and intensely vascularized tumor on the nasal tip.

A 64-year-old man, an alcoholic and smoker since adolescence, without previous comorbidities, sought dermatological care due to the appearance of an erythematous lesion on the face showing progressive growth for seven months, associated with cough with hemoptoic sputum, dyspnea, night fever, and weight loss in the last three months. Upon examination, he had a tumor lesion on the nasal tip, with a lobulated surface, an infiltrated base, which was intensely painful to the touch (Fig. 1). No other findings were found upon physical examination.

**Figure 2:**
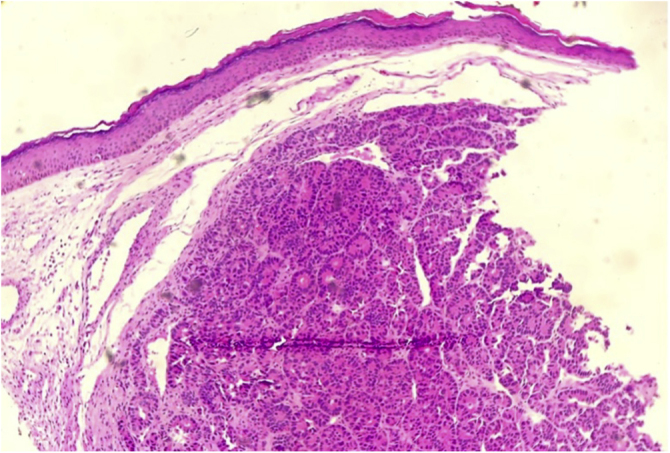
Dermis infiltrated by neoplastic cells arranged in an organoid pattern (Hematoxylin & eosin, ×4).

**Figure 3:**
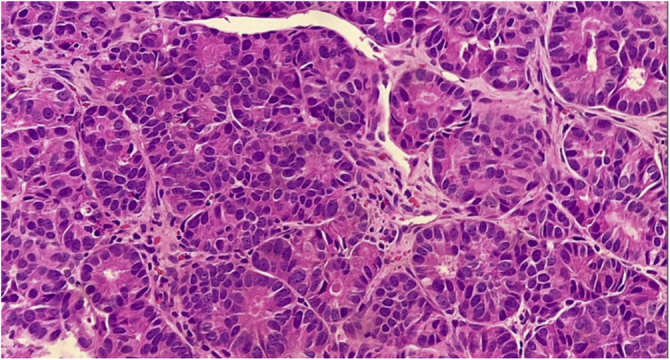
Cells with rounded, hyperchromatic nuclei, eosinophilic cytoplasm, arranged in an organoid pattern (Hematoxylin & eosin, ×40).

**Figure 4:**
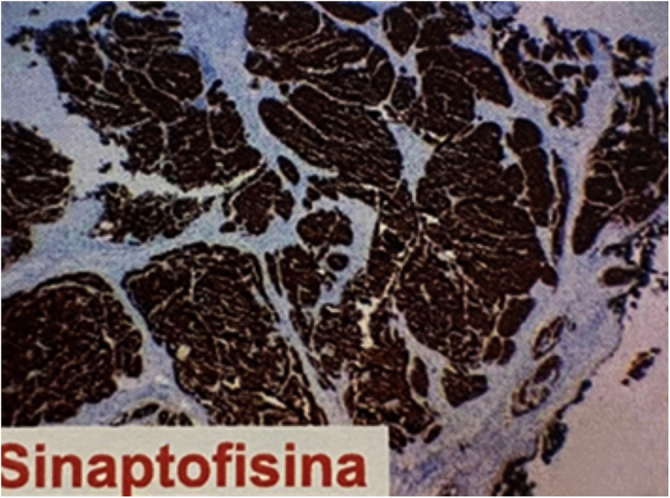
Immunohistochemical study showing strong and diffuse immunoreactivity for synaptophysin.

A biopsy of the skin lesion was performed and the histopathological study showed the dermis infiltrated by a malignant epithelial neoplasm of organoid pattern (Figure 2, Figure 3). Immunohistochemistry showed reactivity with cytokeratin 40, 48, 50, and 50.6 in a Golgi pattern, chromogranin A and synaptophysin (Fig. 4) and was negative for TTF-1 and CK 20, characterizing it as a neuroendocrine tumor. The patient underwent computed tomography of the skull, thorax and total abdomen, which showed an extensively infiltrative pulmonary mass involving the main right bronchus, in addition to multiple lesions suggestive of brain, bone, liver, and adrenal metastasis. Bronchoscopy was performed, but the pulmonary mass biopsy was considered unfeasible due to heavy bleeding during the procedure.

It was concluded that it was a pulmonary carcinoid tumor with metastases to the skin and other parts of the body. The patient was classified as stage IV and referred to the oncologist and died within one month.

## Discussion

Cutaneous metastases (CM) result from the infiltration of the skin by neoplastic cells from distant malignant tumors and can develop by different routes, including hematological and lymphatic dissemination, invasion by contiguity, in addition to iatrogenic implantation.^1^, ^2^ CM develop months to years after the diagnosis of the primary tumor; however, in some cases (37% in men and 6% in women) metastatic cutaneous disease is diagnosed before the underlying internal neoplasia, especially when the metastasis originates from lung, stomach, ovary and kidney cancer.^2^, ^3^ The case reported herein belongs to this rare group of patients where the CM was recognized before the primary lung tumor.

CM usually occur in older individuals and the most affected cutaneous sites are the anterior thoracic and abdominal regions, with the involvement of the face and neck being rare.^1^, ^3^ The frequency of neoplasms produce metastatic cutaneous disease varies according to gender and age group. In adult women, the most common primary tumor sites in decreasing order are as follows: breast, large intestine, lung, and ovary; in adult men, the most prevalent sites follow the sequences: lung, large intestine, oral cavity, and kidney; in children, they comprise neuroblastoma and rhabdomyosarcoma.^1^, ^3^

Determining the origin of the primary tumor, if unknown, can be a difficult and not always possible task. In the case of a carcinoid tumor, with a known histopathological subtype, differentiating whether the skin tumor is primary or secondary is another major challenge for dermatologists and dermatopathologists.^4^ Carcinoid tumors, also called neuroendocrine neoplasms, are derived from enterochromaffin cells and can affect several organs; they are more commonly found in the gastrointestinal tract (65%) and bronchopulmonary tract (25%).^5^ Carcinoid tumor metastases to the skin are rare and, when they occur, the bronchus is the most common primary site.^2^ Clinically, they present as nodules, which are usually multiple, erythematous-violaceous, and mostly asymptomatic; however, there are reports of painful lesions that can progress to ulceration.^5^, ^6^

The histopathological and immunohistochemical analyses help in the definitive diagnosis of carcinoid cutaneous metastases. There is dermal and sometimes subcutaneous infiltration by uniform tumor nests and sheets of cells, with hyperchromatic oval nuclei and scarce cytoplasm, showing immunoreactivity for neuroendocrine markers, including chromogranin, synaptophysin, and low molecular weight cytokeratin. The metastatic cells do not express CK5/6, CK7, CK20, and p63.^2^ The immunoexpression of TTF-1 and CDX2 may be useful in determining the primary site, as the former has shown high sensitivity and specificity for carcinoid tumors originating in the lung, whereas CDX2 expression is highly specific for tumors of gastrointestinal origin.^2^

The differentiation between metastatic carcinoid and primary neuroendocrine tumors of the skin, of which the main representative is Merkel Cell carcinoma (MCC), can also be made through histopathological and immunohistochemical studies. In MCC, subcutaneous involvement is more commonly found, and aggressive neoplasia findings, such as high mitotic index, necrosis, and ulceration are more frequent. Moreover, MCC is typically positive for CK-20, unlike carcinoid tumors of internal origin.^5^, ^6^

In the case described herein, despite the fact that the tumor did not express TTF-1, it was concluded that it was a metastatic cutaneous neuroendocrine carcinoma originating from the lung, considering the immunohistochemical and imaging findings.

In conclusion, although metastatic carcinoid cutaneous neoplasms are rare, they may represent the first manifestation of a neuroendocrine tumor originating in another, yet unknown organ. Therefore, it is worth noting that the high rate of clinical suspicion by dermatologists and their knowledge of histopathological and immunohistochemical findings allow an accurate diagnosis and prompt patient referral for adequate management.

## Financial support

None declared.

## Authors' contributions

Lara Martins Fiorio: Design and planning of the study; drafting and editing of the manuscript; collection, analysis, and interpretation of data; critical review of the literature.

Lucia Martins Diniz: Design and planning of the study; drafting and editing of the manuscript; effective participation in research orientation; critical review of the literature; critical review of the manuscript.

Elton Almeida Lucas: Design and planning of the study; drafting and editing of the manuscript; effective participation in research orientation; critical review of the literature; critical review of the manuscript.

## Conflicts of interest

None declared.
